# Cochlear implant positioning: development and validation of an automatic method using computed tomography image analysis

**DOI:** 10.3389/fsurg.2024.1328187

**Published:** 2024-01-22

**Authors:** Erik H. M. Kemper, Laura M. Markodimitraki, Joëll Magré, Dominique C. Simons, Hans G. X. M. Thomeer

**Affiliations:** ^1^ Department of Otorhinolaryngology & Head and Neck Surgery, University Medical Center Utrecht, Utrecht, Netherlands; ^2^ Education Program Technical Medicine, Leiden University Medical Center, Delft University of Technology & Erasmus University Medical Center Rotterdam, Leiden, Netherlands; ^3^ UMC Utrecht Brain Center, Utrecht University, Utrecht, Netherlands; ^4^ Department of Orthopaedics, University Medical Center Utrecht, Utrecht, Netherlands; ^5^ Technical Medicine, University of Twente, Enschede, Netherlands

**Keywords:** cochlear implant, receiver/stimulator device, algorithm, 3D modelling, computed tomography, temporal bone

## Abstract

The aim of this study was to preoperatively asses the feasibility of drilling a bony recess for the fixation of a cochlear implant in the temporal bone. Even though complications are rare with cochlear implantations, drilling at the site of implantation have resulted in hematoma or cerebrospinal fluid leakage. Mainly in cases with a reduced temporal bone thickness, the risk for complications has increased, such as in paediatric patients.

**Methods:**

An in-house designed semi-automatic algorithm was developed to analyse a 3D model of the skull. The feasibility of drilling the recess was determined by a gradient descent method to search for the thickest part of the temporal bone. Feasibility was determined by the residual bone thickness which was calculated after a simulated drilling of the recess at the thickest position. An initial validation of the algorithm was performed by measuring the accuracy of the algorithm on five 3D models with known thickest locations for the recess. The accuracy was determined by a part comparison between the known position and algorithm provided position.

**Results:**

In four of the five validation models a standard deviation for accuracy below the predetermined cut-off value of 4.2 mm was achieved between the actual thickest position and the position determined by the algorithm. Furthermore, the residual thickness calculated by the algorithm showed a high agreement (max. 0.02 mm difference) with the actual thickness.

**Conclusion:**

With the developed algorithm, a semi-automatic method was created to analyse the temporal bone thickness within a specified region of interest on the skull. Thereby, providing indications for surgical feasibility, potential risks for anatomical structures and impact on procedure time of cochlear implantation. This method could be a valuable research tool to objectively assess feasibility of drilling a recess in patients with thin temporal bones preoperatively.

## 1 Introduction

Born deaf or severely auditory impaired, significantly reduces the societal chances of patients (1). Therefore, a cochlear implant (CI) is a medical solution which has shown to significantly improve auditory capabilities (2).

Implantation of the internal component of the CI consists of insertion of the electrode array in the cochlea, and fixation of the receiver/stimulator (R/S) device on the skull. Although there is extensive literature available for the different surgical techniques of electrode array implantation, definitive evidence regarding optimal fixation techniques of the R/S device is lacking (3). However, migration of the device could lead to surgical complications such as headache, speech processor problems, hematoma, or device failure which can lead to revision surgery (4–9). Two main methods for fixation are being used today by CI surgeons, namely the bony recess and the subperiosteal pocket technique (10, 11). The recommended bony fixation technique requires drilling a recess in the temporal bone to embed the R/S device (12). Usually, a trough, tunnel or overhang is made for protection of the wire. Some CI surgeons use additional sutures or screws to secure the implant. On the other hand, Balkany et al. (13) introduced the more preservative subperiosteal pocket technique in 2009 by which the implant is held in place by the soft tissue of the temporalis muscle and pericranium. Within these two general techniques, a lot of variations exist in execution and use of mesh, sutures or screws for additional fixation (12).

Currently when drilling a bony recess for CI fixation, the location and depth of the recess is chosen perioperatively based on the appropriate distance between transmitter and ear, the shape of the skull, the CI model implanted, and manufacturers guidelines for R/S device placement of at least 2.2 mm (10, 14). However, bone thickness is usually not considered before implantation. The depth of the recess is determined while drilling into the temporal bone during surgery (10).

Even though, cochlear implantation is a relatively safe procedure with few complications, several cases have presented with hematoma or cerebrospinal fluid leakage after compromising the underlying dura mater, vessels and sigmoid sinus at the site of implantation during drilling (15–18). Paediatric patients have a higher chance of exposure of the dura mater due to a thinner temporal bone cortex (19–21). For younger patients it can therefore be questioned whether the temporal bone is thick enough for adequate and safe CI placement using the bony recess fixation method or if an alternative method should be selected preoperatively.

To assess if embedment of the R/S device with sufficient depth according to the guidelines of the manufacturers is possible, an objective preoperative analysis of the temporal bone thickness is needed. Currently, highly accurate and detailed bone segmentations can be calculated from standard computed tomography imaging. These bone segmentations can be used for calculations and measurements in three dimensional space. The results of these three dimensional analysis can provide a preoperative planning to objectively determine if a bony recess can be performed and which structures are at risk during surgery. This feasibility analysis of the surgery can be used to adjust the fixation technique pre-operatively for optimal fixation and further limiting the risk of drilling related complications. Therefore, the aim of this study was to develop and validate an in-house designed algorithm. This algorithm should determine if drilling a bony recess for the fixation of the R/S device is feasible in the temporal bone. This could be used in cases where the thickness of the temporal bone is expected to be inadequate, for example in cases of paediatric cochlear implantation.

## 2 Materials and methods

### 2.1 Ethics

All procedures performed in this study involving human participants were in accordance with the ethical standards of the institutional and/or national research committee and with the 1964 Helsinki declaration and its later amendments or comparable ethical standards. Written informed consent was obtained from the patient whose computed tomography imaging was used in this study.

### 2.2 Study design

#### 2.1.1 Data acquisition

An existing CT scan of a human subject was used (Philips IQon, Netherlands; 236 mA, 120 kV, 0.9 mm slice thickness). Images were stored in DICOM format. Using the segmentation feature in Mimics (version 24.0, Materialise NV, Leuven, Belgium), part of the skull was segmented and reconstructed into a 3D model by thresholding and manual denotation. This 3D model was then imported into 3-Matic (version 16.0, Materialise. Leuven, Belgium). The CI used for this study was the Cochlear CI512, an explanted device from a patient due to hardware failure. Volume data of the CI were acquired by scanning the implant using a 3shape laboratory scanner (3shape, Copenhagen, Denmark). The data was reconstructed into a 3D R/S device model of 24 × 24 × 3.9 mm.

#### 2.1.2 Thickness analysis

To test the feasibility of drilling a recess in the temporal bone, without exposing the dura mater, an in-house designed algorithm was created. Input for the algorithm included a 3D model of the CI recess, a 3D model of the skull and the positions of the right and left proximal external auditory canal and the base of the left orbita. These three landmarks were manually denotated on the CT image and used to automatically determine a region of interest (ROI) on the temporal bone within which the recess could be drilled. This ROI was based on expert opinion (senior CI surgeon, HT) and on manufacturer guidelines.

**Figure 1:**
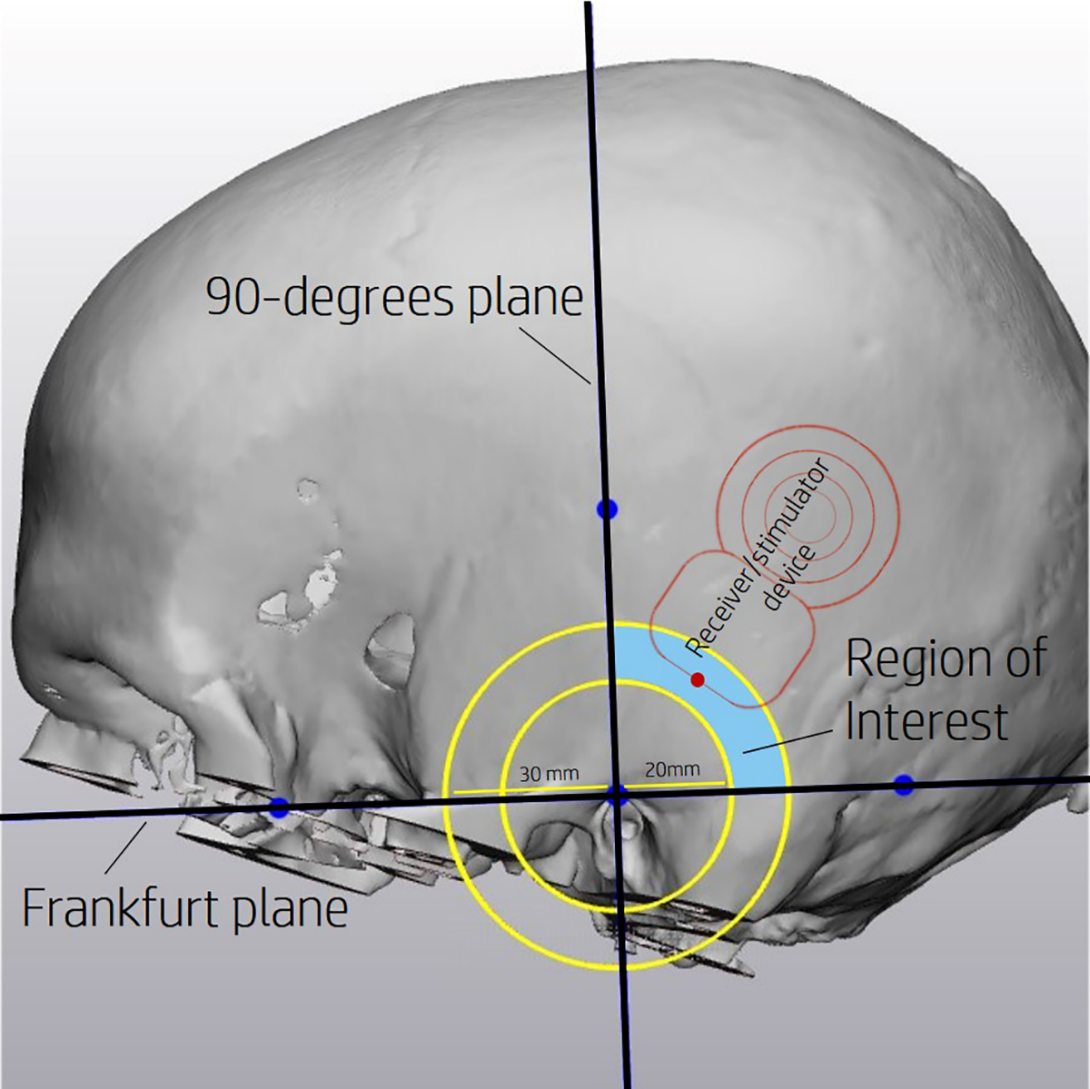
Region of interest (ROI) defined proximal of the Frankfurt plane and posterior of the 90-degrees plane. A minimum distance of 20 mm and a maximum of 30 mm from the external auditory canal further defines the ROI. An example position of the R/S device is depicted by the red outline.

The boundaries of the ROI were defined by several anatomical planes (Figure 1). The region proximal of the Frankfurt plane and posterior of the 90-degrees plane locates the R/S device behind the ear. The minimum distance of 20 mm was needed to provide enough space for the mastoidectomy required to have access to the cochlea. To limit the size of the incision needed to implant the R/S device, a maximum of 30 mm was selected.

**Figure 2:**
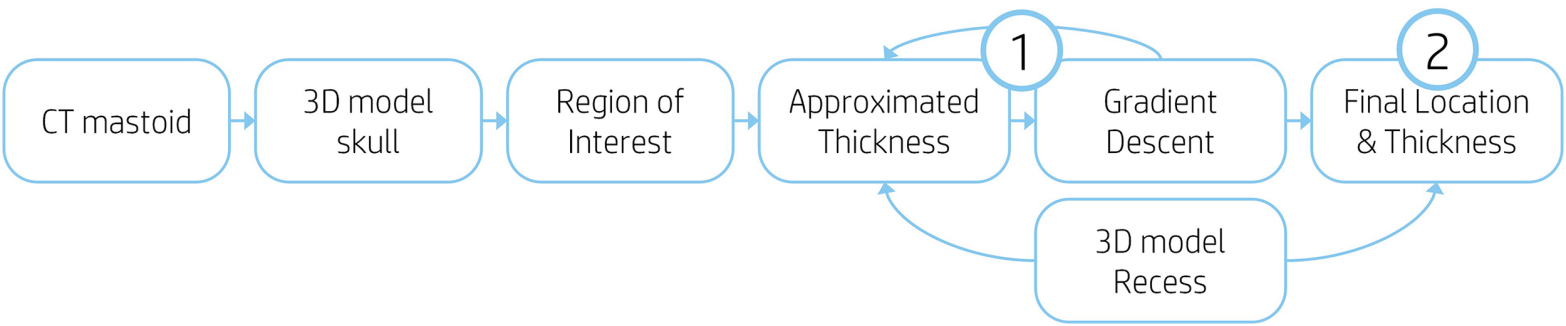
Flow chart of algorithm processes. (1) Iterative search for thicker position. (2) Recess feasibility calculation.

The feasibility was determined by a systematic search performed by the algorithm. This process was performed in two steps. Firstly, a suitable position for the recess was searched iteratively within the ROI, each iteration searching for a thicker position (Step 1 of Figure 2). Secondly, the feasibility of drilling the recess on the final location was determined (Step 2 of Figure 2).

The iterative search within the ROI was performed by a gradient descent method which selects for each iteration a new position based on the direction and intensity of the gradient of the previous iteration. The gradient descent algorithm used a learning rate of 0.7, a step size of 0.8 mm and had a limit of 30 iterations to achieve an optimal location.

**Figure 3:**
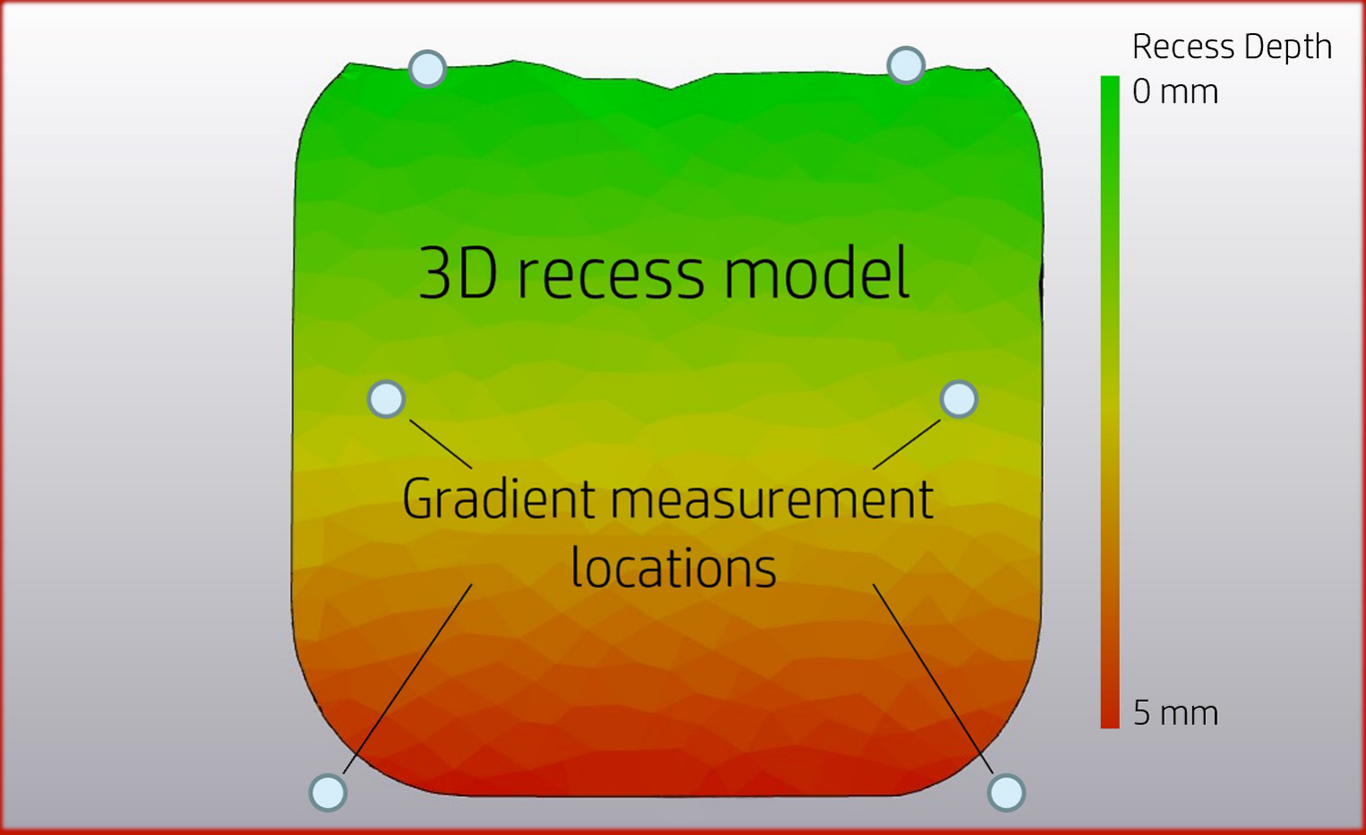
Locations of six reference points in relation to the recess. Colouring representing recess depth related to the original skull surface.

To minimise chance of protrusions by the recess, while limiting the computational power needed for the algorithm, six reference points based on the size of the R/S device model were used to perform thickness measurements (Figure 3). Furthermore, the recess has an increasing depth. To incorporate this gradient, thickness weights were added to each reference point based on the recess depth.

**Figure 4:**
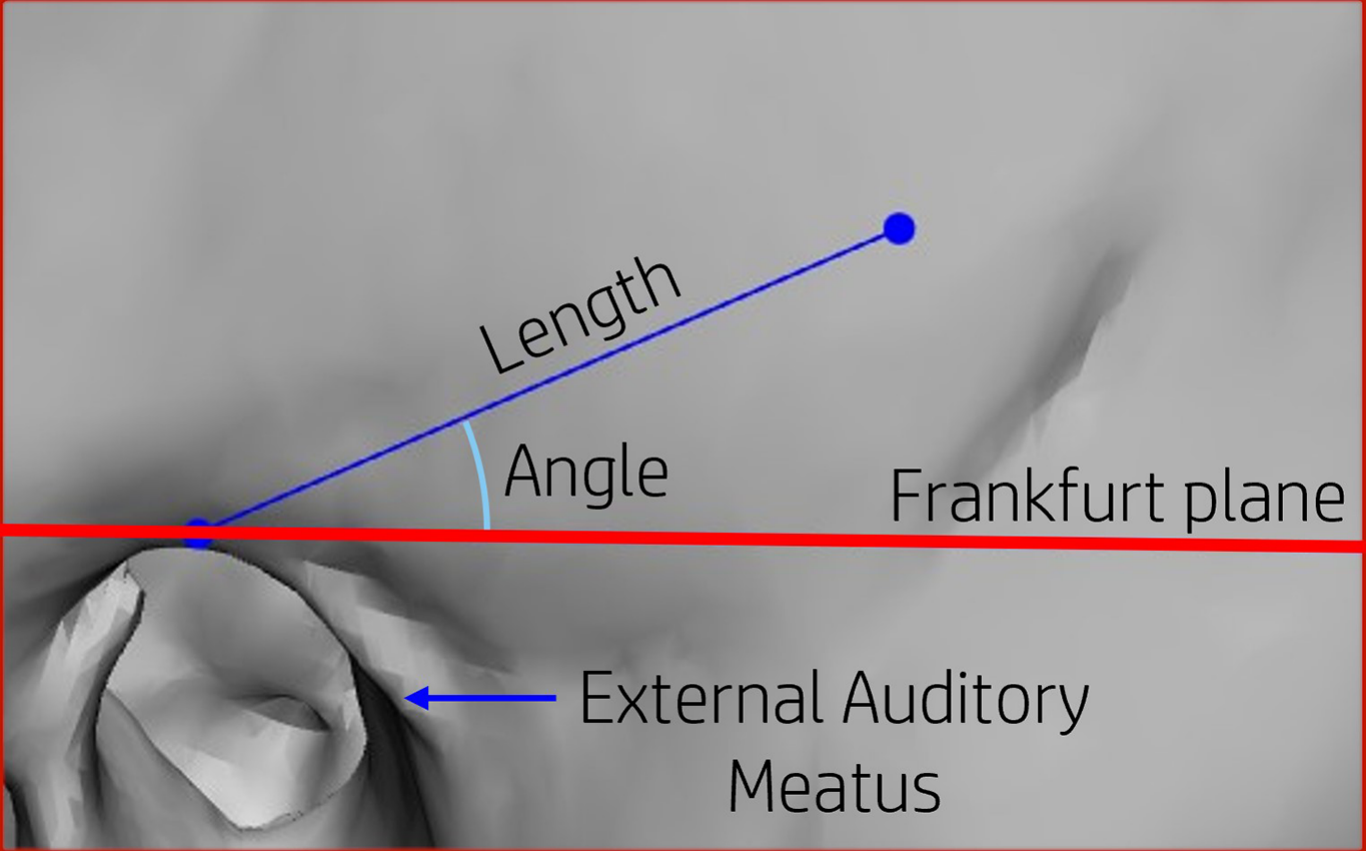
Positions on the skull are defined by a length and angle measured from the proximal point of the external auditory canal in relation to the Frankfurt plane.

The locations of the recess within the ROI were defined by a length and angle (Figure 4). The length is a distance measurement between the external auditory canal and the deepest side of the recess. The angle is measured between the Frankfurt plane and the line created for the length measurement.

**Figure 5:**
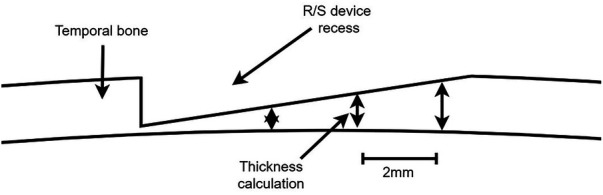
Calculation of R/S device recess feasibility by measuring the thickness of the temporal bone after virtual placement of the recess.

Feasibility of drilling the recess was calculated at the final location determined by the gradient descent method (Figure 5). The residual thickness after recess placement was calculated with a resolution of 2 × 2 mm. If no residual thickness of the skull at any position within the recess was measured, the drilling of a bony recess on the skull was defined as unfeasible.

#### 2.1.3 Validation analysis

To validate the developed algorithm, two validations were performed. First, three 3D spherical models were designed with an insufficient thickness for the recess. The models had a wall thickness of 4.0 mm, 4.5 mm and 4.8 mm respectively. This was done to determine the accuracy of the thickness measurement and to assess if the algorithm would correctly identify models with insufficient bone thickness.

Secondly, 3D models of the skull with a known optimal location for embedment of the R/S device were designed to assess the algorithms ability to identify this area with sufficient bone thickness. The contour of the R/S device was placed within the ROI of the skull and was used to create a local offset. The optimal location for each model was chosen such that different scenarios needed to be solved by the algorithm. These included positions at extreme locations in the ROI. A total of five models from one patient were analysed using the algorithm.

The accuracy between the planned location and the location that was found by the algorithm was calculated by two methods. First a part comparison analysis was performed, resulting in the standard deviation (std) between the two parts. Second the overlapping volume between the planned and algorithmically determined locations was calculated. These calculations were performed by the 3-Matic software (version 16.0, Materialise. Leuven, Belgium).

The optimal locations designed for the 3D models used a R/S device contour 3 mm larger in both surface directions than the actual contour. Therefore, the R/S device could translate 3 mm in the x- and y-direction within the optimal location. Based on the Pythagorean theorem, this results in a standard deviation of 4.2 mm or less to be considered a valid outcome in which the algorithm provided accurate results. The overlapping volume was calculated by dividing the colliding volume with the total volume of the device and multiplying it with 100%.

## 3 Results

The three models with insufficient thickness were correctly identified by the algorithm. The mean thickness measured by the algorithm were 4.02 mm, 4.52 mm and 4.82 mm for the 4.0 mm, 4.5 mm and 4.8 mm models respectively. All with a standard deviation of 0.01 mm between the actual thickness and the measured thickness.

The standard deviations of the second validation analysis ranged from 0.70 to 5.40 mm in the five models (Table 1). For model 1, 3, 4 and 5 standard deviation of less than 4.2 mm was achieved. Model 2 did not achieve a standard deviation below the cut-off value. Overlap volumes of model 4 and 5 exceeded 80%. Model 2 performed worst with a standard deviation of 5.40 and an overlap volume of only 36%. Model 5 performed the best with a standard deviation of only 0.78 and an overlap volume of 87%.

**Table 1:** Results from part comparison analysis and volume overlap for every model.

Model	Std (mm)	Overlap volume (%)
1	2.20	65.12
2	5.40	35.75
3	1.85	69.94
4	1.25	82.45
5	0.78	87.00

## 4 Discussion

In this study we aimed to develop and validate a proof of concept of an algorithm to determine if a recess of the R/S device of a cochlear implant is feasible in the temporal bone ROI. The designed algorithm uses preoperative CT scan imaging and 3D medical software, and is designed to be used by clinicians or research developers. Validation of the algorithm was performed to test the two steps of the algorithm. We first tested the ability of the algorithm to measure bone thickness accurately and detect insufficient bone thickness. Then we assessed if the optimal thickness location could be detected by the algorithm. Five different 3D models with optimal thickness locations were created based on CT imaging of one patient to validate the model. The five created models had sufficient thickness for safe R/S placement during surgery, as described by the algorithm. With a SD of 5.4 mm the determined location by the algorithm for model 2 was slightly outside of the optimal location created by the modelled off-set. While, for models 1, 3, 4 and 5 the determined locations by the algorithm were within the created off-set of the optimal locations.

3D Preoperative analysis of the temporal bone thickness to determine the feasibility of a R/S device recess has been performed before (19, 20). However, standard locations for the recess were used to determine the feasibility, not accounting for differences in anatomy of individuals. The aim of these studies were to calculate a general chance for recess feasibility instead of the personalised analysis provided by the described algorithm. This study was designed to accommodate the needs of the clinician by providing an easy to use, adaptable preoperative analysis method that incorporates operational parameters. Subsequently, the developed algorithm provides more insight in the location of the recess and thereby the relationship with surrounding anatomical structures. The preoperative analysis could provide indications for surgical feasibility, potential risks for anatomical structures and impact on procedure time. In paediatric patients, a higher risk of adverse operational events exists due to their thinner temporal bones' cortex. Prior knowledge on the feasibility of the R/S device recess could reduce the risks of the CI implantation for these paediatric patients by adapting the fixation method of choice preoperatively. The described algorithm is a first step in providing an objective and systematic analysis of the temporal bone thickness and surgical feasibility for cochlear implantation.

The methodology used for the algorithm provides high flexibility for calculation of applicability. The recess model can easily be changed to fit alternative parameters of the ROI, the CI model used, and the recess dimensions. Although the algorithm was designed for thickness feasibility measurement of a R/S device recess, the methodology used in the study could also be applied for other implantable device for which a bone thickness analysis would beneficial (22–24). Furthermore, the time required to perform the analysis is minimal thanks to the limited manual input needed, providing physicians readily available results.

Limitations of this application include the added time and availability of the software needed to perform the analysis. The developed algorithm takes approximately 10 min to apply, however the use of the software applications does require some basic training. Furthermore, the robustness of the current gradient descent algorithm can be improved by addition of a stochastic component.

A limitation of the algorithm validation is the use of a single patient for the model designs. Future clinical implementation studies are suggested to validate the algorithm performance with a diverse set anatomical variations. Of the models used for validation, most of the natural organic features of the skull were retained, thereby potentially introducing confounders. The sample size of the models was still small, validation is needed on a larger scale with actual patients. Furthermore, optimization of the workflow is necessary, before it can be used in clinic. Nevertheless, the proposed algorithm is a proof of concept for the use of automatic thickness measurements in cochlear implantation. Plentiful possibilities exists for further development and optimisation of the algorithm.

With the developed algorithm a semi-automatic method has been created to analyse the temporal bone thickness within a specified ROI. The algorithm provides an easy and flexible way to preoperatively determine if a recess for the R/S device of a cochlear implant can be made. This method could be a valuable tool to objectively assess feasibility of drilling in patients with thin temporal bones for research purposes. For clinical purposes further validation and optimization is needed.

## 5 Resource identification initiative

### 5.1 Tools

Mimics (RRID:SCR_012153)

## Data Availability

The original contributions presented in the study are included in the article/Supplementary Material, further inquiries can be directed to the corresponding author.
